# Antibacterial and Antifungal Activity of Extracts from Five Portuguese Cowpea (*Vigna unguiculata*) Accessions

**DOI:** 10.3390/molecules30112348

**Published:** 2025-05-28

**Authors:** Acácio Salamandane, Mariana Candeias, Susana Lourenço, Emília Joana F. Vieira, Elsa Mecha, Ricardo Gomes, Rosário Bronze, Cátia Nunes, Luisa Brito

**Affiliations:** 1 LEAF—Linking Landscape, Environment, Agriculture and Food Research Center, Associate Laboratory TERRA, Instituto Superior de Agronomia, Universidade de Lisboa, Tapada da Ajuda, 1349-017 Lisboa, Portugal; salamandane@gmail.com (A.S.); susanaineslourenco@gmail.com (S.L.); vieiraemilia@gmail.com (E.J.F.V.); 2 Faculdade de Ciências de Saúde, Universidade Lúrio, Campus Universitário de Marrere, Nampula 4250, Mozambique; 3 iBET—Instituto de Biologia Experimental e Tecnológica, Av. da República, Apartado 12, 2781-901 Oeiras, Portugal; emecha@ibet.pt (E.M.); ragomes@ibet.pt (R.G.); mbronze@ibet.pt (R.B.); 4 Instituto de Tecnologia Química e Biológica António Xavier, Universidade Nova de Lisboa, Av. da República, 2780-157 Oeiras, Portugal; 5 Unidade Estratégica de Investigação e Serviços de Biotecnologia e Recursos Genéticos, Instituto Nacional de Investigação Agrária e Veterinária, Instituto Público, Avenida da República, Quinta do Marquês, 2780-157 Oeiras, Portugal; catia.soares@iniav.pt

**Keywords:** cowpea (*Vigna unguiculata*) leaf extracts, quercetin, foodborne pathogenic bacteria, *Penicillium expansum*, minimum inhibitory concentration (MIC), minimum bactericidal concentration (MBC)

## Abstract

Under the principles of the circular economy and sustainability, consumers, the food industry and health authorities have interest in new natural food preservatives to prevent foodborne diseases and increase produce shelf life. This work aimed to evaluate the antimicrobial properties of cowpea plant extracts. Grain, pod and leaf extracts from five Portuguese cowpea accessions were characterized in terms of their phenolic content. The values of minimum inhibitory concentration (MIC) and minimum bactericidal concentration (MBC) were determined against pathogenic and non-pathogenic bacteria, as well as on post-harvest pathogenic filamentous fungi. In general, the phenolic content of pod extracts was higher than that of grains and leaves, although leaf extracts had the highest content of a broad-spectrum antibacterial flavonoid, quercetin. Grain extracts exhibited no detectable antimicrobial activity. In contrast, leaf and pod extracts from all five accessions generally displayed bactericidal effects. For bacteria, pod extracts showed MICs ranging from 5.1 to 87.7 mg/mL and MBCs from 20.3 to 87.7 mg/mL. Leaf extracts showed the most promising results, with MICs and MBCs ranging from 1.1 to 9.1 mg/mL. The results against fungi were not so expressive; nevertheless, *P. expansum* was inhibited by 9 L leaf extract even if at a higher concentration (MIC = 18 mg/mL) than those obtained for bacteria. The Portuguese variety Fradel (1E) showed very promising antibacterial activity, with leaf extracts showing low MBC values (from 2.3 to 9.1 mg/mL). The obtained results indicate that cowpea pods and leaves have antimicrobial properties and could potentially be used as a source of compounds for food preservation.

## 1. Introduction

Cowpea (*Vigna unguiculata*) is a leguminous plant widely cultivated in tropical and subtropical regions, particularly in Africa, Asia and Latin America [1]. However, cowpea can also be found in Mediterranean countries such as Greece, Italy, Spain and Portugal [2,3]. As for Portugal, there are no statistical data regarding the production of cowpea, but this crop is known to be cultivated in the regions of Beiras, Alentejo and Trás-os-Montes, being mainly used for consumption in human diets [2]. The agronomical and environmental values of pulses are deeply connected, since these crops can be used in different farming systems, enriching the soil by trapping atmospheric nitrogen, all the while having a very low nutritional and hydric demand [2,4], serving as food for humans but also fodder for cattle feeding [5,6,7]. Cowpea seeds, leaves and pods are sources of protein, dietary fiber, vitamins and minerals. For these reasons, cowpea is very important in food security strategies in developing countries [8,9]. In addition to its nutritional benefits, the potential of cowpea as a source of bioactive compounds with antimicrobial properties has been highlighted, offering promising applications in food preservation and public health [10,11,12,13,14,15].

Foodborne pathogens such as *Listeria monocytogenes* and *Salmonella enterica* (namely, serovar Thyphimurium) continue to pose significant challenges to food safety worldwide. These microorganisms are responsible for foodborne illnesses that affect millions of people every year, leading to hospitalizations, deaths and substantial economic losses. Thus, effective strategies to control bacterial contamination in food systems are essential to ensure food safety and reduce public health risks [16,17].

These challenges result in a growing interest in exploring plant-based biocides as alternatives to synthetic preservatives [18,19,20,21]. Plants have long been recognized as a source of bioactive compounds with antimicrobial properties [22,23,24,25,26,27]. These compounds, often called phytochemicals, can inhibit the growth of microorganisms or eliminate them completely. A variety of plant extracts have therefore been studied for their potential antimicrobial activities, including those derived from herbs, spices and fruits [18,19,20], and legumes are no exception [28]. Cowpea has shown promising potential due to its phytochemical profile, which includes phenolic compounds, flavonoids, tannins, saponins and peptides [23,29]. Phenols vary from simple, low-molecular-weight compounds with a single aromatic ring to complex tannins and polyphenol derivatives. They are categorized based on the number and arrangement of their carbon atoms into flavonoids—including flavonols, flavones, flavan-3-ols, anthocyanidins, flavanones and isoflavones—and non-flavonoids, such as phenolic acids, hydroxycinnamates and stilbenes. These compounds are often found conjugated with sugars and organic acids. Phenols are known to have diverse biological activities, including antibacterial, antifungal, antioxidant and anti-inflammatory effects [27]. Given the growing global demand for natural food preservatives and the need for effective strategies to combat foodborne pathogens, research on extracts from the cowpea plant is quite promising [18,19,20].

This study aimed to investigate the antimicrobial properties of cowpea plant extracts on different microorganisms, including pathogenic and non-pathogenic bacteria (Gram-positive and Gram-negative) and filamentous fungi.

## 2. Results

### 2.1. Phenolic Content of the Extracts

Table 1 shows the phenolic contents of the extracts of grains, leaves and pods of the five cowpea accessions analyzed. The content of phenolic compounds (expressed in gallic acid equivalents per 100 g of dry matter) in cowpea varied according to the part of the plant and the accession. In the pods, this content varied between 405.9 and 877.2, in the leaves it varied between 95.4 and 279.4 and in the grain, it varied between 64.0 and 263.7 mg/100 g. The accessions 9L and 13B stood out for the highest concentration of phenolic compounds in the pods. In the grains, the accessions 3E and 13B presented higher contents, while, in the leaves, the landrace 3E presented the highest value. In the leaves, the landrace 3E presented the highest value, while in the grains the effect of seed coloring on the phenolic content is clear, with the white/cream-colored seeds (1E and 9L) with the lowest values and the clay and black seeds with the highest values (3E, 13B and 5V, respectively).

### 2.2. Metabolic Diversity of Cowpea Extracts

Table 2 presents a tentative identification of phenolic compounds present in the extracts.

Phenolic content differed among plant tissues (Table 2). Grain extracts present the highest amount of identified compounds compared with pods and leaves. Pods lack several flavonoids in complex with sugars, like quercetin-3-arabinoside, quercetagetin-7-O-glucoside, kaempferol-3-gentiobioside and kaempferol-3-gentiobioside and also procyanidin B1 and B2. Leaf extracts lack all those and many others, particularly phytoestrogens like genistein and coumestrol.

**Figure 1:**
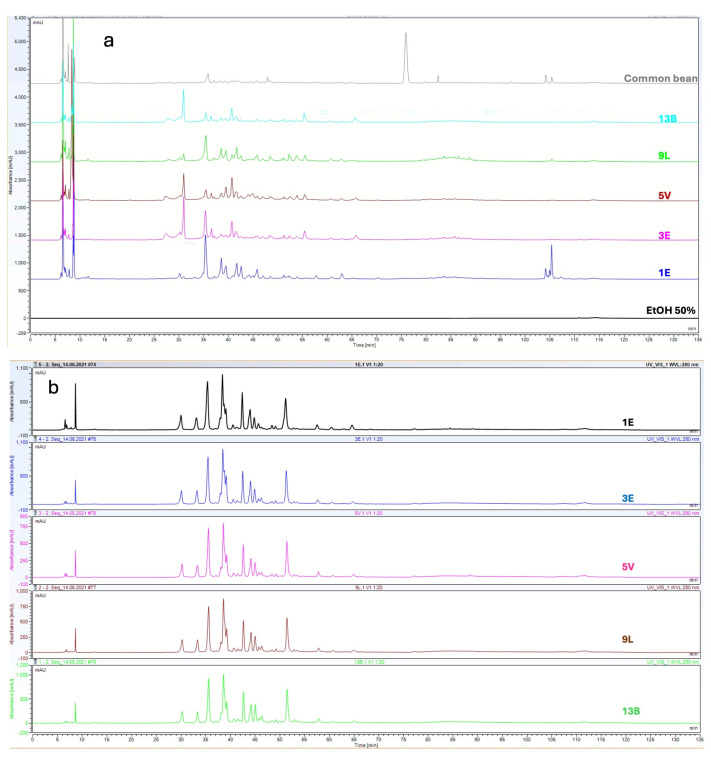
Representative HPLC chromatograms of cowpea samples. (**a**) HPLC−280 nm chromatograms from grain extracts. (**b**) HPLC−280 nm chromatograms from pod extracts.

**Figure 2:**
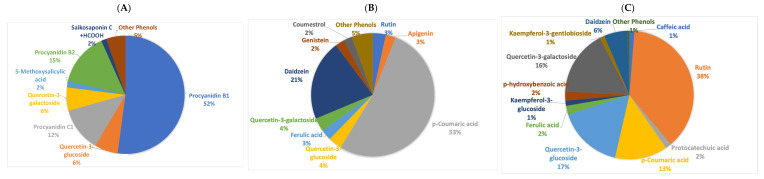
Pie chart indicating the percentage distribution of the most abundant compounds in cowpea (**A**) grain, (**B**) pod and (**C**) leaf extracts.

The extracts from the three tissues were quite different in terms of relative percentage of phenolic compounds (Figure 1). In the grains, the three most abundant compounds were three procyanidins. In the pod extracts, p-coumaric acid was the most abundant compound followed by daidzein, a phytoestrogen, while in the leaf extracts, quercetin was the most abundant phenolic compound (Figure 2).

### 2.3. Antifungal Activity of Cowpea Extracts from Variety 9L (Guarda Do Douro)

Grain and pod extracts from landrace 9L did not show antifungal activity. *A. flavus* and *A. niger* did not show any type of inhibition with 9L leaf extract. However, the MIC of leaf extract of landrace 9L for *P. expansum* was 18.1 mg/mL.

### 2.4. Antibacterial Activity

The extracts from grains from the five accessions tested did not show antibacterial activity against the four bacterial strains tested.

#### 2.4.1. MIC and MBC of Extracts of Pods and Leaves

Pod and leaf extracts did show antibacterial activity against the four bacterial strains tested (Table 3 and Table 4).

In general, the MIC values of pod extracts differed with the tested bacteria and cowpea accessions. Landraces 3E (Sátão/Viseu) and 5V (Vila Maior/Viseu) stood out for presenting better inhibitory capacity (lower MIC values) (Table 3). The landrace 3E (Sátão/Viseu) pod extract showed promising results against the pathogenic bacteria *L. monocytogenes* and *Salmonella*. However, Gram-positive bacteria were generally more susceptible than Gram-negative bacteria.

The MIC values of leaf extracts varied with the bacteria but did not vary greatly from accession to accession (Table 3). Variety 1E (Fradel) and landrace 3E (Sátão/Viseu) were those that presented the best capacity to inhibit the bacteria under study (Table 3). Variety 1E stood out as it presented the lowest MIC value for *Salmonella*. Likewise, Gram-positive bacteria were more susceptible than Gram-negative bacteria. Pod extract from landrace 13V showed no inhibition, even at the highest concentration tested.

The MBC values of extracts of pods and leaves for the four bacterial strains are shown in Table 4.

As expected, the MBC values of cowpea extracts vary with bacteria and cowpea accession under test. Landrace 5V had the pod extract with the lowest MBC values (Table 4). Also, in general, it was found that the two Gram-positive bacteria were more susceptible to pod and leaf extracts than the two Gram-negative bacteria (Table 3 and Table 4). For Gram-positive bacteria, the MBC of pod extracts ranged from 20.3 to 62.7 mg/mL, and the MBC of leaf extracts ranged from 1.1 to 4.5 mg/mL. Regarding Gram-negative bacteria, the MBC values of pod extracts varied from 29.0 to 87.7 mg/mL and the MBC of leaf extracts was 9.1 mg/mL, except for the leaf extract of variety 1E against *Salmonella* that was 4.5 mg/mL (Table 4). Regarding Gram-positive bacteria, in general the non-pathogenic bacterium, *L. innocua*, was more susceptible than *L. monocytogenes*. The same does not happened with Gram-negative bacteria. *Salmonella*, in general, was more susceptible than the nonpathogenic *E. coli* (Table 3 and Table 4).

In 22 of the 36 cases analyzed, the MBC obtained corresponded to the MIC (MBC/MIC = 1) (Table 5).

#### 2.4.2. Mean Logarithmic Reduction in Bacterial Cell Viability

The knowledge of the logarithmic reduction in bacterial cell viability associated with MIC and MBC values is useful because it supports the antimicrobial properties of the cowpea extracts analyzed. Table 6 shows the respective average logarithmic reductions (log CFU/mL) with the extracts in MIC and in MBC. The logarithmic reductions associated with MIC ranged between 1.6 log CFU/mL and 7.4 log CFU/mL. The logarithmic reduction associated with MBC ranged between 3.9 log CFU/mL and 7.4 log CFU/mL.

Regarding the foodborne pathogenic bacterium *L. monocytogenes*, the more effective extracts, with total logarithmic reduction (Table 6) and (MIC = MBC) (Table 5) were pod extract from landrace 5V (29 mg/mL) (Table 3 and Table 4), and pod extract from landrace 13B (62.1 mg/mL) (Table 3 and Table 4) and, particularly, leaf extract (2.3 mg/mL) (Table 3 and Table 4) from landrace 13B.

Regarding the foodborne pathogenic bacterium *Salmonella* Thyphimurim, the four tested leaf extracts from accessions 1E, 3E, 9L and 13B, were effective with total logarithmic reduction (Table 6) and (MIC = MBC) (Table 5), particularly variety 1E (4.5 mg/mL) (Table 3 and Table 4).

## 3. Discussion

Plant extracts in general are essential for advancing microbiological control in a circular economy, promoting sustainability, safety and efficiency. Their use not only addresses microbial challenges, but also contributes to environmental conservation, economic empowerment and public health, supporting the transition to greener and more resilient systems. Moreover, some plant extracts have proven effective in controlling microbial growth in various food products, limiting the spread of foodborne pathogens and reducing antibiotic resistance [30]. Several studies have demonstrated the potential of phenolic-rich plant extracts and pure plant phenolics to inhibit the growth of undesirable microorganisms in diverse food matrices, highlighting their value as natural antimicrobial agents [14,18,31].

Several studies demonstrated that the phenolic content of cowpea varies according to the country of origin [32,33]. The variability obtained is due to the genetic diversity of the grain [34], causing not only differences in the total phenolic content, but also in the phenolic composition [35]. The high variability in composition could also be influenced by factors such as the method and conditions under which the extraction was carried out [36].

In this work, the content of phenolic compounds in Portuguese cowpea varied according to the part of the plant and the accession. Pods showed higher content than grains and leaves. The landraces 9L (Lardosa/Castelo Branco) and 13B (Guarda) stood out for the highest concentration of phenolic compounds in the pods. The landraces 3E (Sátão/Viseu) and 13B (Guarda) presented the highest content in the grains. Landrace 3E (Sátão/Viseu) presented the highest value in the leaves. When analyzing the composition of the extracts from the different plant tissues, seeds are richer in procyanidins, pods have a higher concentration of p-coumaric acid and daidzein, a phytoestrogen, while leaves have higher concentrations of quercitins (Figure 2). The antimicrobial activity of an extract does not depend solely on their most abundant molecules, but on potential interactions and synergistic effects among compounds. Nevertheless, quercitins are described to be an efficient broad spectrum antibacterial flavonoid [37]. Procyanidins have also been described to have antimicrobial and anti-biofilm activity especially against Gram-positive bacteria [38], while p-coumaric acid has only moderate antimicrobial activity [37]. In soybean, it has also been described that isoflavones, genistein and daidzein have relevant antibacterial activity [39].

Grain extracts have been reported as a source of antimicrobial activity against foodborne pathogens [14,18,31]. However, the extracts of grains from the accessions analyzed did not show any type of activity against bacteria or fungi. This is in contrast to results observed in study of Abdel-Shafi et al. [31], where cowpea seed globulins demonstrated antibacterial activity against *Escherichia coli* and *Staphylococcus aureus*, with MICs in the range of 5–10 mg/mL. The lack of activity in our grain extracts may be attributed to the lower concentration or absence of bioactive proteins or peptides, or differences in the extraction solvents and targeted phytochemicals. While procyanidins are known for their antioxidant properties, their antimicrobial activity is not consistently observed across all extracts. Factors such as extraction methods, compound composition, and degree of polymerization play crucial roles in determining their efficacy [39]. Extracts of pod and leaves of variety 9L were used against fungi, but only *P. expansum* was inhibited with leaf extract, although at a higher concentration that those required for bacteria. According to some authors [40,41,42] both *Aspergillus* and *Penicillium* may show susceptibility to cowpea extracts [42,43,44], although within the same genus and species, susceptibility may vary [45].

The antibacterial activity of the pod and leaf extracts varied depending on the accession of cowpea and the bacterial species. Pod extracts from variety 3E showed the lowest MIC values for the pathogenic bacteria *Salmonella* Thyphimurium and *L. monocytogenes* (20.4 mg/mL). The observed activity may be more due to daidzein and genistein than to p-coumaric acid which is described in the literature to have a more restricted antibacterial activity [37]. Lenny and Rizky [10], reported that leaf methanolic extracts from *V. unguiculata* inhibited *S. aureus* at concentrations around 12.5 mg/mL. Our leaf extracts, especially from variety 1E (Fradel), exhibited MICs as low as 1.1 mg/mL against *L. monocytogenes*, which is considerably lower than previously reported values, highlighting their superior potency and novelty. Nevertheless, leaf extracts showed even more promising results, in accordance with the high antibacterial activity described for quercitins [37]. Variety 1E and landrace 3E showed the lowest MIC values for the pathogenic bacteria *Salmonella* Thyphimurium (4.5 and 9.1 mg/mL) and *L. monocytogenes* (1.1 mg/mL). These MIC values are notably lower than those found in other legume-based studies. For example, Cetin-Karaca and Newman [24] documented MICs ranging from 10 to 50 mg/mL for phenolic-rich legume extracts against similar bacteria. This supports the uniqueness of the Portuguese cowpea accessions tested in this study.

In general, Gram-positive bacteria were more susceptible to pod and leaf extracts than Gram-negative bacteria. This difference in susceptibility can be explained by cell wall structure. Gram-negative bacteria have an outer membrane composed of lipopolysaccharides, which can impair the interaction of phenolic compounds with the peptidoglycan layer [46,47]. In addition, the lipophilic characteristics of certain compounds in the extracts can reduce their affinity for the lipid membrane of Gram-negative bacteria, reducing their effectiveness [48]. Previous studies [10,14], corroborate that Gram-negative bacteria are less susceptible to phenolic compounds than Gram-positive bacteria, due to differential interaction with the cell wall.

In 22 of the 36 cases analyzed (extracts versus bacterial species), the MBC obtained corresponded to the MIC (MBC/MIC = 1) and in 34 of the 36 cases analyzed the MBC/MIC ratio is ≤2, which means that, in general, the Portuguese cowpea pod and leaf extracts tested may be considered bactericidal [49]. This high frequency of bactericidal behavior (MBC/MIC ≤ 2 in 94.4% of cases) strengthens the case for practical application. Similar studies using cowpea seed protein fractions [30,31] showed more limited bactericidal effects. Therefore, the bactericidal profile of our leaf and pod extracts represents a novel and promising antimicrobial potential. This shows that the extracts may be promising for application as natural food preservatives or disinfectants. Moreover, at MBC, leaf extract from variety 1E (Fradel) resulted in total reduction in viable cells of the four bacterial species tested, while extracts from landrace 13B resulted in total reduction in viable cells of three of the four species used, including the two pathogens.

The results obtained showed than the antibacterial activity of the leaf extracts is higher than the pod extracts. However the phenolic content of the pod extracts is higher than the leaf extracts (two to eight times higher in pods than in leaves). The grains, that showed the same order of magnitude of phenolic content as leaves, had no antimicrobial activity at the concentrations tested. This higher antimicrobial activity found in cowpea leaf extracts may be, at least in part, related to the high amount of quercetin. These molecules are known to have broad-spectrum antimicrobial activity [37]. It is important to note that the results observed were variable depending on the cowpea accession in study, which can be explained by different phenolic and peptide profiles. Also the type of extraction (here, hydro-alcoholic) can influence the yield of the bioactive compounds obtained. It is therefore necessary to conduct further studies in order to unveil the mechanisms of action of the bioactive compounds present in cowpea and their direct or indirect effect on human health. This is particularly important due to their potential application as functional ingredients and additives in foodstuffs.

## 4. Methods

### 4.1. Plant Extracts

The extracts were obtained from one Portuguese cowpea variety and four Portuguese landraces, corresponding to references 1E (commercial variety Fradel), 3E (Sátão/Viseu), 5V (Vale Pedro, Vila Maior/Viseu), 9L (Lardosa/Castelo Branco) and 13B (Guarda do Douro). The plant material used for the extraction of phenolic compounds (grains, pods and dried leaves) was ground in a laboratory mill (Cyclone Mill Twister, Retsch, Germany) at 14,000 rpm, obtaining particles with a diameter of 0.8 mm. The ground material was stored at −20 °C until it was used for the extraction of phenolic compounds.

**Figure 3:**
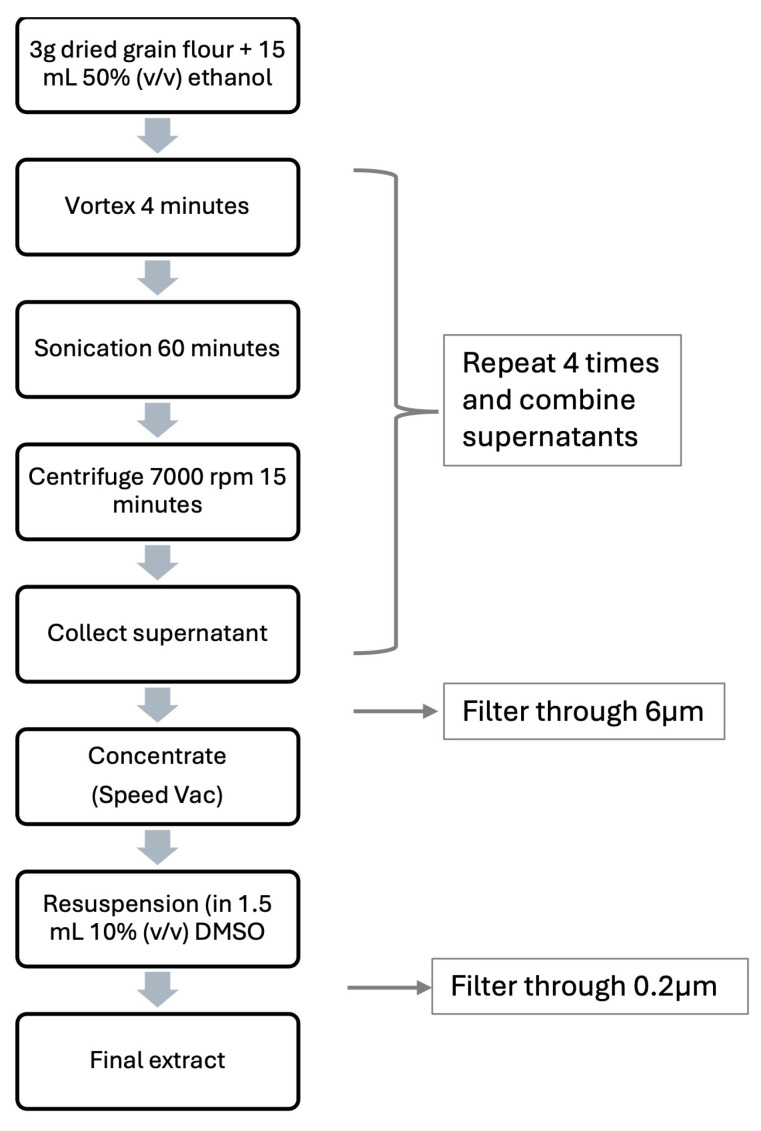
Schematic representation of the extraction method.

The extraction of phenolic compounds was performed according to the protocol of Lin et al. (2008) [30], with minor modifications. Briefly, 15 g of previously ground plant material was extracted with a 50% (*v*/*v*) aqueous ethanol solution in a 1:4 (*m*/*v*) ratio, followed by vortexing for 4 min and sonication (Digital Ultrasonic Cleaner, Argo Lab, Carpi MO, Italy) for 60 min at 25 °C. The mixture was centrifuged at 7000 rpm (Sorvall ST 16, Thermo Scientific, Waltham, MA, USA) for 15 min. The supernatant was filtered through 90 mm, 6 μm pore diameter filter paper (Grade 1F, Munktell, Sweden). The final extracts were dried (Acid-Resistant CentriVap Concentrator, Labconco, Kansas, MO, USA) and within a day, diluted in 10% (*v*/*v*) of dimethyl sulfoxide (DMSO) (Sigma-Aldrich Chemie GmbH, Taufkirchen, Germany). The extracts were then filtered with a 25 mm CA filter with a pore diameter of 0.20 μm (CHROMAFIL Xtra PTFE-20/25, Macherey-Nagel, Germany) and stored at −20 °C until use (Figure 3).

### 4.2. Total Phenolic Content (TPC)

TPC was determined by the Folin–Ciocalteu method with modifications. Briefly, Folin–Ciocalteu reagent (0.100 mL) was added to 3.5 mL of extracts previously diluted according to the fraction and variety. After 3 min, 0.400 mL of sodium carbonate solution (35%, *w*/*v*) was added, and after one hour the absorbance was measured against water, in a Spectrophotometer DU-70 (Beckman^®^, Brea, CA, USA), at 725 nm. Gallic acid was used as the external standard in a concentration range of 1 to 6 mg/L of gallic acid. Results were expressed in milligram of gallic acid equivalents (mg GAE) per g of dry weight (DW).

### 4.3. In Vitro Antioxidant Activity

The Oxygen Radical Absorbance Capacity (ORAC) assay was applied to evaluate antioxidant capacity of cowpea whole flour towards peroxyl radicals. The assay was carried out following a modified method described by Ou et al. (2001) [50], in order to measure the ability of antioxidant species, present in the sample, to inhibit Fluorescein (FL) oxidation catalyzed by 2,2′-Azobis(2-amidinopropane) dihydrochloride (AAPH)—generated peroxyl radicals (ROO). The reaction mixture included 6.3 × 10^−8^ M FL, 1.28 × 10^−2^ M AAPH (prepared in 75 mM PBS, pH 7.4) and the diluted sample, in a total volume of 1.8 mL. The reaction started by addition of AAPH to the mixture, placed in a 10 mm wide fluorescence cuvette at 37 °C. Fluorescence emitted by the reduced form of FL was measured and recorded every 1 min at the emission wavelength of 515 nm and excitation wavelength of 493 nm (fluorescence spectrophotometer with thermostatic bath, model Cary Eclipse, Varian Ltd., Surrey, UK) for a period of 30 min. PBS was used as blank and 1, 5, 12.5, 25 and 50 M Trolox solutions as control standards. For ORAC analysis, only the whole flour extracts were analyzed. All samples, including blank and controls, were analyzed in triplicate. Final ORAC values were calculated using a regression equation established between Trolox concentration and the net area under FL decay curve. Data were expressed in micromoles of Trolox equivalents antioxidant capacity (TEAC) per g of seed’s dry weight (DW).

### 4.4. Characterization of Phenolic Compounds Through Quadrupole Time-of-Flight (QTOF) Mass Analyzers

In a Mass Spectrometer X500 QTOF (Agilent, Santa Clara, USA), Sciex, equipped with a Turbo Ion Spray at 500 °C, TOFMS and TOFMS/MS scan types in negative and positive modes were applied during 20.5 min with an injection volume of 5 µL and a flow rate of 0.4 mL/min. For separation, 0.1% formic acid in Milli-Q^®^ water (eluent A) and 0.1% formic acid in ace-tonitrile (eluent B) were applied in gradient mode: 10% (B) at the beginning, ramping to 99% B at 13 min and remaining during 2 min in the 99% (B). At 16 min, the elution returned to the initial conditions remaining at 10% (B) during the last 4 min in a XBridge BEH C18, 130 Å, 3.5 µm, 2.1 × 150 mm column, at 30 °C. The data were acquired using SciEX software (Triple Quad™ 7500 LC-MS/MS System—Q, https://sciex.com/products/software, accessed on 22 November 2024). Commercial standards (catechin, caffeic acid, chlorogenic acid, rutin, quercetin, kaempferol, apigenin, protocatechuic acid, procyanidin B1, p-coumaric acid, quercetin-3-glucoside, procyanidin C1, ferulic acid, kaempferol-3-glucoside, quer-cetin-3-arabinoside and vanillic acid) were analyzed under the same conditions. Identical retention times and mass accuracies (lower than 10 ppm) were applied as compounds ID confirmation parameters. The untargeted analysis was conducted using the available libraries (e.g., NIST 2017) analyzing the library score (>50).

### 4.5. Data Processing, Identification and Relative Quantification of Compounds

The collected data were analyzed using the Finnee2016 toolbox for untargeted metabolomics analysis (Erny, Acunha, Sim’o, Cifuentes, & Alves, 2016) [51]. The final compounds list (defined by *m*/*z* values) was aligned according to the retention time for further statistical analysis. The final excel file was exported to MetaboAnalyst (version 4.0) freely available at https://www.metaboanalyst.ca/ (accessed on 22 November 2024), for statistical analysis and metabolites selection (Chong et al., 2018) [52]. The data were log transformed and pareto-scaled. Multivariate analysis by partial least square-discriminant analysis (PLS-DA) allowed to select the most relevant compounds.

The compounds were identified using the Compound Discoverer software, version 2.1, (Thermo Scientific™, MA, USA). The relative quantification was conducted by comparison of the percent area of individual compounds considering the different analyzed cowpea accessions.

### 4.6. Bacteria and Fungi Used

Strains of Gram-positive pathogenic (*Listeria monocytogenes*) and non-pathogenic (*Listeria innocua*) and of Gram-negative pathogenic (*Salmonella* Typhimurium) and non-pathogenic (*Escherichia coli*) food-borne bacteria were used (Table 7).

Three species of postharvest pathogenic filamentous fungi were also used, namely, *Penicillium expansum*, *Aspergillus flavus* and *Aspergillus niger* (Table 7).

### 4.7. Evaluation of the Antimicrobial Activity of the Extracts

The antimicrobial activity of cowpea extracts (beans, pods and leaves) was assessed by determining the minimum inhibitory concentration (MIC) and the minimum bactericidal concentration (MBC). The MIC value corresponding to the lowest concentration of the extract that prevented visible microbial growth, after 24 h of incubation at 37 °C (bacteria), or after 48 h of incubation at 25 °C (fungi).

For bacteria, a logarithmic reduction of more than 1 log (90% reduction in microbial viability) was also used as criterion for confirming the MIC value [50,53,54]. The MBC was calculated as the minimum concentration of the extract that induces microbial death, after removable of the extract [55,56] based on logarithmic reduction equal to or greater than 3 log (99.9% reduction in microbial viability), according to previous publications [34,35,36,37,38]. Bacterial cells and fungal conidia were also exposed to serial twofold dilutions of DMSO (10% (*v*/*v*) to confirm the absence of inhibition).

#### 4.7.1. Antifungal Activity

##### Preparation of the Conidial Suspensions

Fungi were used in active growth (exponential phase), after inoculation of the center of Potato Dextrose Agar (PDA) (Sigma-Aldrich Chemie GmbH, Taufkirchen, Germany). plates with 5 mm peripheral mycelium discs. The mycelial discs were from a stock maintained in sterilized water at 4 °C. After inoculation, the plates were incubated at 25 °C for the necessary time (ca. 5 days) for the mycelial growth to almost reach the periphery of the PDA plates. Fungal conidial suspensions were prepared from 5-day cultures on PDA by flooding the culture with 0.01 % (*v*/*v*) Tween 80 (Sigma-Aldrich Chemie GmbH, Taufkirchen, Germany) and dislodging the spores with the aid of a sterilized stainless-steel spreader. The resulting suspension was then filtered, in a sterilized funnel, through three sheets of sterilized gauze and collected in a 50 mL Falcon tube. The conidia in the suspension were counted in a hemocytometer (Neubaeur Improved Hirschmann Techcolor) on a light microscope (Leica, Wetzlar, Germany), to adjust the spore stock concentration to 1 × 10^6^ spores/mL in sterile distilled water, kept at 4 °C, until use for MIC determinations.

##### Determination of the MIC of the Extracts from Landrace 9L Against Fungi

The evaluation of the MICs of the extracts against the phytopathogenic fungi, was performed, basically according to the microdilution broth method, but in Potato Dextrose Broth (PDB) medium (Sigma-Aldrich Chemie GmbH, Taufkirchen, Germany), using 96 microwells plates (NunclonTM, Roskilde, Denmark). Briefly, after producing serial twofold dilutions of each stock solution of the respective plant extract in each microplate, 100 µL of the suspension of fungal spores (1 × 10^6^ spores mL^−1^) was added in each well in a total volume of 200 µL. Spores that were not exposed to the plant extract at any time and PDB that was not inoculated with the spores at any time were controls for the viability of the inoculum and the sterility of the PDB medium. The MIC was defined as the lowest concentration of the plant extract that prevented visible growth of the fungi after an incubation period of 48 h at 25 °C. For each plant extract, the determination of MIC value was performed with two replicates, in two independent trials.

#### 4.7.2. Antibacterial Activity

##### Preparation of the Inocula

The bacterial cultures were inoculated onto TSA-YE (Oxoid, Hampshire, UK) plates and incubated at 37 °C in order to obtain isolated colonies. Bacterial cells taken from single isolated colonies on overnight incubated TSA-YE plates were used to inoculate 10 mL of Mueller Hinton broth (AES Laboratoire, Bruz, France) and incubated overnight at 37 °C. The concentration of the inoculum was always confirmed by triplicate plating on TSA-YE and incubation at 37 °C for 24 h before counting colonies.

##### Determination of the MIC and MBC of the Extracts Against Bacteria

The determination of MIC values for the four bacterial strains used was performed in MH, at 37 °C, in triplicate, at least in two independent trials, using 96 microwells plates (NunclonTM, Roskilde, Denmark), basically according to the microdilution broth method. Briefly, after producing serial twofold dilutions of the extracts stock solutions in each microplate, 100 μL of inoculum was added in each well in a total volume of 200 µL (1 × 10^6^ CFU mL^−1^). Bacterial cells that were not subjected to extracts at any time and MH that was not inoculated at any time were controls for viability of the inoculum and sterility of the culture medium. The wells were sealed with 50 μL of sterile paraffin (Labsolve, Odivelas, Portugal) in order to prevent evaporation. After 24 h incubation, the MIC and the MBC were also determined by direct inoculation of 0.1 mL of each suspension, from the microplates, onto TSA-YE plates. The remaining suspensions were, respectively, decimal diluted and 100 μL aliquots were spread onto TSA-YE plates. This allowed to determine the logarithmic reduction in cell viability. For each isolate and extract, two biological replicates with two technical replicates each were performed. If the MBC/MIC ratio was ≤2, the test agent was considered bactericidal. If the MBC/MIC ratio > 2, the agent was considered bacteriostatic [37].

## 5. Conclusions

This work emphasized the antimicrobial properties of leaf extracts of Portuguese cowpea accessions, with a view to using it as natural food preservatives or as part of food disinfectants. Furthermore, considering the concept of the circular economy, it would be interesting for future studies to continue exploring extracts obtained from cowpea leaves, since they are undervalued parts of the plant, although, as discussed, they have a high content of bioactive compounds. Our results suggest the potential use of cowpea leaf extracts, particularly the Portuguese commercial variety 1E (Fradel), as natural antimicrobial agents. This meets the goals of a circular economy and therefore deserves further investigation. Future research should focus on the isolation and structural characterization of the specific compounds responsible for the antimicrobial activity observed, particularly quercetin and isoflavones like daidzein and genistein. In addition, evaluating the efficacy of these extracts in real food systems and under different processing conditions will be critical to assess their practical application as natural food preservatives.

## Figures and Tables

**Table 1:** Phenolic content (mg gallic acid equivalents/100 g dry mass) and antioxidant activity (μmole of Trolox equivalents antioxidant capacity (TEAC) per g of seed’s dry weight) of cowpea extracts from grains, leaves and pods.

	Phenolic Content (mg Gallic Acid Equivalents/100 g Dry Mass)			Antioxidant Activity (μmole of Trolox Equivalents Antioxidant Capacity per g of Seed’s Dry Weight)
Cowpea Reference	Grains	Leaves	Pods	Grains
**1E**—commercial Fradel	70.5 ± 15.5	117 ± 9.1	516.7 ± 12.3	38.6 ± 11.7
**3E**—Sátão/Viseu	263.7 ± 12.1	279.4 ± 37.8	569.2 ± 16.0	163.8 ± 4.6
**5V**—Vale Pedro, Vila Maior/Viseu	219.9 ± 4.7	nd	405.9 ± 17.0	130.5 ± 8.6
**9L**—Lardosa/Castelo Branco	64.0 ± 5.2	95.4 ± 16.1	877.2 ± 29.7	29.7 ± 7.6
**13B**—Guarda do Douro	257.5 ± 28.2	158.1 ± 32.3	868.7 ± 47.4	125.7 ± 10.6

nd—not determined.

**Table 2:** Tentative identification, by Mass Spectrometer X500 QTOF, of phenolic compounds present in grain, pod and leaf cowpea extracts.

Compound	Family; Class	Formula	Found at Mass	Grains	Pods	Leaves
Catechin	Flavonoids; Flavanol	C_15_H_14_O_6_	291.088	x	x	
Caffeic acid	Phenolic acids; Hydroxycinnamic acid	C_9_H_8_O_4_	181.0513	x	x	x
Rutin	Flavonoids; Flavonol glycoside	C_27_H_30_O_16_	611.1643	x	x	x
Quercetin	Flavonoids; Flavonol	C_15_H_10_O_7_	303.0508	x	x	x
Kaempferol	Flavonoids; Flavonol	C_15_H_10_O_6_	287.057	x	x	x
Apigenin	Flavonoids; Flavonol	C_15_H_10_O_5_	271.0617	x	x	x
Protocatechuic acid	Phenolic acids; Hydroxybenzoic acid	C_7_H_6_O_4_	155.0353	x	x	x
Procyanidin B1	Flavonoids; Proanthocyanidin (dimer)	C_30_H_26_O_12_	579.1503	x		
p-Coumaric acid	Phenolic acids; Hydroxycinnamic acid	C_9_H_8_O_3_	165.0555	x	x	x
Quercetin-3-glucoside	Flavonoids; Flavonol glycoside	C_21_H_20_O_12_	465.1038	x	x	x
Epicatechin	Flavonoids; Flavanol	C_15_H_14_O_6_	289.0723	x	x	
Procyanidin C1	Flavonoids; Proanthocyanidin (trimer)	C_45_H_38_O_18_	867.2143	x	x	
Ferulic acid	Phenolic acids; Hydroxycinnamic acid	C_10_H_10_O_4_	195.0663	x	x	x
Kaempferol-3-glucoside	Flavonoids; Flavonol glycoside	C_21_H_20_O_11_	449.1102	x	x	x
Quercetin-3-arabinoside	Flavonoids; Flavonol glycoside	C_20_H_18_O_11_	435.0918	x		
Vanillic acid	Phenolic acids; Hydroxybenzoic acid	C_8_H_8_O4	169.0508	x	x	
p-hydroxybenzoic acid	Phenolic acids; Hydroxybenzoic acid	C_7_H_6_O3	139.0399	x		x
Syringic acid	Phenolic acids; Hydroxybenzoic acid	C_9_H_10_O5	199.0609		x	
Quercetin-3-galactoside	Flavonoids; Flavonol glycoside	C_21_H_20_O_12_	465.1038	x	x	x
Phloretin	Dihydrochalcones; Flavonoid-like polyphenol	C_15_H_14_O_5_	275.0967	x		
5-Methoxysalicylic acid	Phenolic acids; Methoxylated hydroxybenzoic acid	C_8_H_8_O4	167.0355	x		
Procyanidin B2	Flavonoids; Proanthocyanidin (dimer)	C_30_H_26_O_12_	579.1503	x		
Quercetagetin-7-O-glucoside	Flavonoids; Flavonol glycoside	C_21_H_19_O_13_^−^	479.0854	x		
Kaempferol-3-gentiobioside	Flavonoids; Flavonol diglycoside	C_27_H_30_O_16_	609.1478	x		x
Quercetin 3-O-β-D-glucose-6′-acetate	Flavonoids; Acylated flavonol glycoside	C_23_H_22_O_13_	505.1008	x	x	
5,7,3′,4′,5′-Pentahydroxyflavone	Flavonoids; Flavonol	C_15_H_12_O_7_	301.0364	x		
Daidzein	Isoflavonoids; Isoflavone	C_15_H_10_O_4_	253.0514	x	x	x
(+)-Abscisic acid	Terpenoids; Sesquiterpenoid	C_15_H_20_O_4_	263.1294	x	x	
Genistein	Isoflavonoids; Isoflavone	C_15_H_10_O_5_	269.0463	x	x	
Coumestrol	Coumestans; Phytoestrogen	C_15_H_8_O_5_	267.0308	x	x	
2-Hydroxymyristic acid	Fatty acids; Hydroxy fatty acid (not a phenoplic compound)	C_14_H_28_O_3_	243.1972	x		
Apigeninidin cation	Anthocyanidins; Flavylium cation	C_15_H_11_O_4_^+^	255.2331	x	x	
5,7-Dimethoxyapigeninidin cation	Anthocyanidins; Methoxylated flavylium cation	C_17_H_15_O_4_	283.2651	x		

**Table 3:** MIC values (mg/mL) of pod and leaf extracts of the five cowpea accessions for the four bacterial strains.

Bacteria	Pod Extracts					Leaf Extracts			
	Accessions								
	1E	3E	5V	9L	13B	1E	3E	9L	13B
	MIC (mg/mL)								
*Listeria innocua* (Gram-positive and non-pathogenic)	12.9	14.2	5.1	21.9	−	1.1	1.1	2.3	1.1
*Listeria monocytogenes* (Gram-positive and pathogenic)	36.9	20.4	29.0	31.4	62.1	1.1	1.1	2.3	2.3
*Escherichia coli* (Gram-negative and non-pathogenic)	51.7	56.9	10.1	87.7	46.5	9.1	9.1	9.1	9.1
*Salmonella enterica Thyphimurium* (Gram-negative and pathogenic)	73.9	20.4	29.0	62.7	62.1	4.5	9.1	9.1	9.1

(−) Absence of inhibition with the highest concentration tested.

**Table 4:** MBC values (mg/mL) of pod and leaf extracts of the five cowpea accessions for the four bacterial strains.

Bacteria	Pod Extracts					Leaf Extracts			
	Accessions								
	1E	3E	5V	9L	13B	1E	3E	9L	13B
	MIC (mg/mL)								
*Listeria innocua* (Gram-positive and non-pathogenic)	25.8	28.5	20.3	43.9	−	2.3	2.3	2.3	1.1
*Listeria monocytogenes* (Gram-positive and pathogenic)	39.6	40.7	29.0	62.7	62.1	2.3	1.1	4.5	2.3
*Escherichia coli* (Gram-negative and non-pathogenic)	51.7	56.9	40.6	87.7	46.5	9.1	9.1	9.1	9.1
*Salmonella Thyphimurium* (Gram-negative and pathogenic)	−	40.7	29.0	62.7	62.1	4.5	9.1	9.1	9.1

(−) Absence of inhibition with the highest concentration tested.

**Table 5:** MBC/MIC ratio of pod and leaf extracts of the five cowpea accessions for the four bacterial strains.

Bacteria	MBC/MIC Ratio for the Extracts of the Five Cowpea Accessions								
	1E		3E		5V	9L		13B	
	Pod	Leaf	Pod	Leaf	Pod	Pod	Leaf	Pod	Leaf
*Listeria innocua* (Gram-positive and non-pathogenic)	2	2	2	2	4	2	1	^b)^	1
*Listeria monocytogenes* (Gram-positive and pathogenic)	1	2	2	1	1	2	2	1	1
*Escherichia coli* (Gram-negative and non-pathogenic)	1	1	1	1	4	1	1	1	1
*Salmonella* Thyphimurium (Gram-negative and pathogenic)	^a)^	1	2	1	1	1	1	1	1

^a)^ It was not possible to determine the MBC (reduction < 3 log); ^b)^ Absence of inhibition with the highest concentration tested.

**Table 6:** Mean logarithmic reduction in viable bacterial cells with MIC and MBC of cowpea pod and leaf extracts.

*Bacteria*	Pod Extracts										Leaf Extracts							
	Accessions																	
	1E		3E		5V		9L		13B		1E		3E		9L		13B	
	MIC	MBC	MIC	MBC	MIC	MBC	MIC	MBC	MIC	MBC	MIC	MBC	MIC	MBC	MIC	MBC	MIC	MBC
	∆ (Log Initial Number of CFU/mL—Log Final Number of CFU/mL)																	
*Listeria innocua* (Gram-positive and non-pathogenic)	2.0	6.2	2.1	4.1	1.9	3.9	1.6	4.6	-	-	2.6	6.5	2.7	6.5	4.5	4.5	4.3	4.3
*Listeria monocytogenes* (Gram-positive and pathogenic)	5.3	5.3	2.4	**6.8**	**6.8**	**6.8**	2.4	**6.8**	**6.8**	**6.8**	1.8	**6.5**	3.3	3.3	2.8	**7.2**	**6.5**	**6.5**
*Escherichia coli* (Gram-negative and non-pathogenic)	5.3	4.8	**6.3**	**6.3**	2.0	**6.3**	**6.3**	**6.3**	3.7	3.7	**7.2**	**7.2**	**7.2**	**7.2**	5.2	5.2	**7.4**	**7.4**
*Salmonella Thyphimurium* (Gram-negative and pathogenic)	1.9	-	2.0	**6.7**	**6.7**	**6.7**	5.7	5.7	5.1	5.1	**7.4**	**7.4**	**7.4**	**7.4**	**7.4**	**7.4**	**7.4**	**7.4**

∆ Values in bold—total logarithmic reduction; (-) Absence of inhibition with the highest concentration tested.

**Table 7:** Microorganisms used in this study.

Type	Reference	Species
Bacteria	CBISA3008 =NCTC11288 =ATCC33090	*Listeria innocua*
	CBISA3001 =NCTC11994 = CECT4032	*Listeria monocytogenes* serovar 4b
	CBISA3965	*Escherichia coli* B
	CBISA3969 =ATCC14028	*Salmonella enterica* serovar Typhimurium
Filamentous fungi	Unnamed internal collection	*Penicillium expansum*
		*Aspergillus flavus*
		*Aspergillus niger*

CBISA—Colecção de Bactérias do Instituto Superior de Agronomia; NCTC—National Collection of Type Cultures; ATCC—American Type Culture Collection; CECT—Colección Española de Cultivos Tipo.

## Data Availability

All data supporting the findings of this study are available within the article.
